# Production of xylose through enzymatic hydrolysis of glucuronoarabinoxylan from brewers’ spent grain

**DOI:** 10.1186/s40643-022-00594-4

**Published:** 2022-10-04

**Authors:** Lilia C. Rojas-Pérez, Paulo C. Narváez-Rincón, M. Angélica M. Rocha, Elisabete Coelho, Manuel A. Coimbra

**Affiliations:** 1 Departamento de Ingeniería Química, Facultad de Ingeniería, Universidad Ean, 110221 Bogotá D.C., Colombia; 2 Departamento de Ingeniería Química y Ambiental, Facultad de Ingeniería, Universidad Nacional de Colombia, 111321 Bogotá D.C., Colombia; 3 Departamento de Química, Universidade de Aveiro, 3810-193 Aveiro, Portugal

**Keywords:** Brewers’ spent grain, Glucuronoarabinoxylan, Arabinoxylan, Xylan saccharification, Synergism, Xylanolytic enzymes, Enzymatic hemicellulose hydrolysis

## Abstract

Xylose is an abundant bioresource for obtaining diverse chemicals and added-value products. The production of xylose from green alternatives like enzymatic hydrolysis is an important step in a biorefinery context. This research evaluated the synergism among four classes of hydrolytic purified enzymes—endo-1,4-β-xylanase, α-l-arabinofuranosidase, β-xylosidase, and α-d-glucuronidase—over hydrolysis of glucuronoarabinoxylan (GAX) obtained from brewers’ spent grain (BSG) after alkaline extraction and ethanol precipitation. First, monosaccharides, uronic acids and glycosidic-linkages of alkaline extracted GAX fraction from BSG were characterized, after that different strategies based on the addition of one or two families of enzymes—endo-1,4-β-xylanase (GH10 and GH11) and α-l-arabinofuranosidase (GH43 and GH51)—cooperating with one β-xylosidase (GH43) and one α-d-glucuronidase (GH67) into enzymatic hydrolysis were assessed to obtain the best yield of xylose. The xylose release was monitored over time in the first 90 min and after a prolonged reaction up to 48 h of reaction. The highest yield of xylose was 63.6% (48 h, 40 ℃, pH 5.5), using a mixture of all enzymes devoid of α-l-arabinofuranosidase (GH43) family. These results highlight the importance of GH51 arabinofuranosidase debranching enzyme to allow a higher cleavage of the xylan backbone of GAX from BSG and their synergy with 2 endo-1,4-β-xylanase (GH10 and GH11), one β-xylosidase (GH43) and the inclusion of one α-d-glucuronidase (GH67) in the reaction system. Therefore, this study provides an environmentally friendly process to produce xylose from BSG through utilization of enzymes as catalysts.

**Graphical Abstract:**

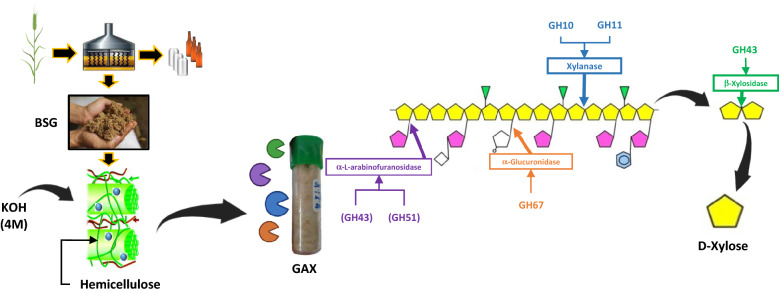

## Introduction

In the context of biorefining, the utilization of hemicellulose as the second most abundant sugar component of lignocellulosic biomass is highly desired, hemicellulose can be transformed into oligosaccharides composed of xylose or in monomeric sugars, mainly xylose. Xylooligosaccharides (XOS) are considered prebiotic compounds presenting technical and health claims (Poletto et al. 2020; Swart et al. 2020). Xylose derived products including xylitol, ethanol, isobutanol, lactic acid or lipid and its derivatives by biological technologies (Long et al. 2022). Agricultural crop and industrial residues have been explored to obtain hemicellulose between them corncob, corn stover, wheat straw and the main byproduct of beer production known as brewers’ spent grain (BSG). BSG is a complex biomass composed of starch (2–13%), cellulose (13–21%), hemicellulose (19–42%) and lignin (12–16%), with a high protein content (19–30%) (Lynch et al. 2016; Rojas-Pérez et al. 2022).

Methods for converting plant material into monomeric sugars are required for their further use as feedstocks in the production of a range of value-added products, these procedures comprise pretreatment and enzymatic hydrolysis (EH) steps (Gonçalves et al. 2022). Pretreatment of lignocellulosic biomass is crucial prior to enzymatic hydrolysis. Various pretreatment options have been reported to fractionate, solubilize, hydrolyze, and separate cellulose, arabinoxylan (AX), and lignin components (Saha 2003). Specifically, AX can be extracted using water or hydrothermal pretreatment (Kaur et al. 2020), alkaline pretreatment (Pérez-Flores et al. 2019), ultrasound-assisted extraction (Reis et al. 2015) and microwave (Coelho et al. 2014). The AX of BSG are usually extracted by a well-established sequentially procedure using solutions of KOH with increasing concentration of 0.5 M, 1 M, and 4 M follow for ethanol precipitation (Mandalari et al. 2005; Vieira et al. 2014). Efficient hydrolysis of hemicellulose is a major challenge to the enzymatic conversion of lignocellulose (Liu et al. 2021). The conversion of AX into monosaccharides or derived compounds can be achieved by enzymatic or chemical catalysis, with the latter using metal oxides (Naidu et al. 2018). Although AX are readily hydrolyzed to monosaccharides by acid treatment, enzymatic hydrolysis is preferable for the industrial upgrading of AX, mainly because enzymatic hydrolysis permits specific, controlled modifications of the reaction that prevent the generation of undesirable byproducts and generally facilitate more environmentally friendly processes (Sørensen et al. 2007a, b; Meyer et al. 2009).

AX are characterized by a backbone of (β1 → 4)-linked d-xylopyranosyl residues partially substituted with single units of α-l-arabinofuranosyl at positions 2, 3, or both (Moreirinha et al. 2020). Phenolic acids, such as ferulic and *p*-coumaric acids, are esterified to arabinofuranosyl residues (Mandalari et al. 2005). Coelho et al. (2016) also identified other substituents in AX from BSG, such as uronic acid, methylated uronic acid, and an acetyl group, showing the presence of GAX in BSG. Due to the complex branching and heterogeneous composition of GAX, enzymatic degradation requires the synergistic action of depolymerizing and debranching enzymes. Depolymerization relies on endo-1,4-β-xylanases (EC 3.2.1.8) and β-xylosidases (EC 3.2.1.37). Endo-1,4-β-xylanases randomly attack the 1,4-β bonds within the xylan backbone to generate unsubstituted or branched xylo-oligosaccharides (XOS) and xylobiose, whereas β-xylosidases attack the non-reducing ends of short-chain xylo-oligosaccharides and xylobiose to release xylose (Sørensen et al. 2007a, b). Debranching enzymes mainly include α-l-arabinofuranosidases (EC 3.2.1.55), α-d-glucuronidases (EC 3.2.1.139), ferulic acid esterases (EC 3.1.1.73), and/or acetyl xylan esterases (EC 3.1.1.72).

Commercial cellulase–hemicellulase mixtures, enzyme extract from microorganisms, high purity enzymes or the combination between them had been probed in different hemicellulose fraction extracted from lignocellulosic biomass. Xiros et al. (2008) for example, evaluated a multi-enzymatic system (xylanase, endoglucanase, cellobiohydrolase, β-glucosidase, α-l-arabinofuranosidase, acetyl esterase, and feruloyl esterase) from *N. crassa* over BSG and alkali pre-treated BSG, found that enzymatic hydrolysis of alkali pre-treated BSG was increased about 50% compared with non-pretreated material and the released sugars (glucose, xylose, arabinose) from pre-treated BSG using the enzyme extract from *N. crassa* achieved a yield about 50% of total pentose content and about 60% of total glucose in the material. On the other hand, prolonged treatment (24 h with a 50:50 mixture of Celluclast^®^ 1.5 L and Ultraflo^®^ L at 50 °C, pH 5) in water-soluble wheat arabinoxylan achieve a xylose release of 62 wt% (Sørensen et al. 2005)*.* Another work from Sørensen et al (2007a, b) found that 114.5% of xylose was released from water-soluble wheat AXs with a “minimal” enzyme 20:20:20:40 mixture of Abf II (α-l-arabinofuranosidase *H. insolens* GH43 family), Abf III (α-l-arabinofuranosidase *M. giganteus* GH51 family), Xyl III (endo-1,4-β-xylanase *H. insolens* GH10 family), and β-xyl (β-xylosidase *T. reesei* GH3 family), although the yield above 100% of the “theoretical maximum” presumably resulted from the degradation of arabinose and xylose during the acid hydrolysis. Mccleary et al. (2015) determined empirically an optimal enzyme mixture that contained β-xylanase (1300 U/mL), β-xylosidase (200 U/mL), *B. adolescentis* α-l-arabinofuranosidase (300 U/mL), *U. maydis* α-l-arabinofuranosidase (75 U/mL), and *A. niger* α-l-arabinofuranosidase (170 U/mL) as high purity enzymes over water-soluble wheat flour arabinoxylans (WAXs) and found a yield of 90% of l-arabinose and d-xylose release, although the addition of more enzymes did not increase the yield, suggesting the presence of other groups in the arabinoxylan, such as ferulic acid, which prevent complete hydrolysis of WAX to monosaccharides. Newly, Long et al. (2022) evaluated the hydrolysis of corncob arabinoxylans with low (CAX1) or high (CAX2) branching degrees reported that a new arabinofuranosidase EpABF62A of the GH62 family combined with a GH10 xylanase, a GH43 β-D-xylosidase and a GH67 α-glucuronidase released 75.0% or 64.5% xylose from CAX1 or CAX2, respectively.

The aim of this research was to evaluate different strategies based on the addition of one or two families of enzymes—endo-1,4-β-xylanase (GH10 and GH11) and α-l-arabinofuranosidase (GH43 and GH51)—cooperating with one β-xylosidase (GH43) and one α-d-glucuronidase (GH67)—over hydrolysis of GAX fraction (4 M KOH and ethanol precipitation) from BSG monitoring the time in the first 90 min and after a prolonged reaction up to 48 h of reaction, to obtain xylose as monomeric sugar.

## Materials and methods

### Extraction of GAX from BSG

A freeze-dried GAX from BSG were used as the raw material. This fraction was obtained from a supernatant solution by centrifugation from cellulosic residue after 4 step in a sequential alkaline extraction (4 M KOH + 5 mM Na_2_S_2_O_5_), subsequently acidified to pH 3 with citric acid to permit the precipitation of BSG proteins, the fraction soluble in citric acid, which contain the GAX were finally separated with ethanol precipitation, following the procedure described by Vieira et al. (2014).

### Structural analysis of GAX extract from BSG

#### Monosaccharide composition analysis

Monosaccharides were released from cell wall polysaccharides by prehydrolysis with 0.2 mL of 72% H_2_SO_4_ (*w*/*w*) for 3 h at room temperature, followed by 2.5-h hydrolysis with 1 M of H_2_SO_4_ at 100 ℃ (Selvendran et al. 1979). Neutral sugars were analyzed to determine their alditol acetate content using gas chromatography flame ionization detection (GC-FID) (Blakeney et al. 1983; Harris et al. 1988). Duplicate hydrolysis was performed for all fractions.

#### Uronic acids

Uronic acids were quantified using the 3-phenyl phenol colorimetric method and the calibration curve for galacturonic acid (200 mg/mL) (Selvendran et al. 1979; Coimbra et al. 1996).

#### Glycosidic-linkage composition of the polysaccharide fraction

The glycosidic-linkage composition was determined using gas chromatography quadrupole mass spectrometry (GC–qMS) for the partially methylated alditol acetates, as described by Coelho et al. (2014) and Reis et al. (2015), using CH_3_I as a methylating reagent. Duplicate methylation was performed for all fractions.

### Hydrolysis of GAX from BSG

#### Enzymes

Six pure enzymes were obtained from Megazyme International (Bray, County Wicklow, Ireland). Their families, substrates, and main characteristics are described in Table 1.

**Table 1:** Summary of enzymes characteristics

Enzyme	CAZy family	Microorganism	Substrate	T optima (℃)	pH optima
endo-1,4-β-Xylanase (EC 3.2.1.8)	GH11	*Neocallimastix patriciarum*	Endo-hydrolysis of (1,4)-β-d-xylosidic linkages in xylans	50	6.0
	GH10	*Cellvibrio japonicus*		60	5.0
α-l-Arabinofuranosidase (EC 3.2.1.55)	GH43	*Bifidobacterium adolescentis*	Highly specific hydrolysis of α-1,3-linked l-arabinofuranose residues from doubly substituted d-xylosyl or l-arabinosyl residues of arabinoxylans and branched arabinans, respectively	50	6.0
	GH51	*Aspergillus niger*	Hydrolysis of α-1,2- and α-1,3-linked l-arabinofuranose residues from arabinoxylans and branched arabinans. Hydrolyses α-1,5-linked arabino-oligosaccharides at a much lower rate	40	4.0
exo-1,4-β-d-Xylosidase (EC 3.2.1.37)	GH43	*Selenomonas ruminantium*	Hydrolysis of (1,4)-β-d-xylans and xylo-oligosaccharides to remove successive d-xylose residues from non-reducing termini	50	5.0
α-d-Glucuronidase (EC 3.2.1.139)	GH67	*Geobacillus stearothermophilus*	Hydrolysis of the α-1,2 glycosidic bond between d-glucuronic acid or its ether 4-O-methyl-d-glucuronic acid from the terminal non-reducing d-xylose residues of xylo-oligosaccharides (aldo-uronic acids) and xylan	70	7.0

#### Enzyme combinations

The reaction mixture was adjusted to a concentration of 1 g/L of GAX (4 M KOH) from BSG in 100 mM of sodium succinate buffer (pH 5.5) at temperature of 40 °C. A fixed dose of 0.2 mg of enzyme-protein/g potential GAX mixture was added, which contained N. patriciarum endo-1,4-β-xylanase GH11 (1300 U/mL), C. japonicus endo-1,4-β-xylanase GH10 (500 U/mL), B. adolescentis α-l-arabinofuranosidase GH43 (300 U/mL), A. niger α-l-arabinofuranosidase GH51 (170 U/mL), S. ruminantium β-d-xylosidase GH43 (200 U/mL), and G. stearothermophilus α-d-glucuronidase GH 67 (200 U/mL). For pH (5.5) and enzyme doses, the research adopted the values reported by Mccleary et al. (2015). The reaction temperature (40 °C) was established as a balance of the optimal temperatures for the six pure enzymes evaluated, considering both the optimal values reported in the enzyme datasheets and the references in the literature for this type of enzyme reaction (Xiros et al. 2011; Rasmussen et al. 2012; Mccleary et al. 2015). Table 2 shows the nomenclatures and definitions of the enzymes used in each test.

**Table 2:** Definition of the addition of enzymes and nomenclature of the tests carried out to evaluate the synergistic effect

Assay	Mixture enzyme	Enzymes present in the assay	Abbreviations
I	All	All enzymes	all
II	Without one enzyme	All enzymes devoid of endo-1,4-β-xylanase (GH11)	all-X_GH11_
III		All enzymes devoid of endo-1,4-β-xylanase (GH10)	all-X_GH10_
IV		All enzymes devoid of α-l-arabinofuranosidase (GH43)	all-A_GH43_
V		All enzymes devoid of α-l-arabinofuranosidase (GH51)	all-A_GH51_
VI	Without two enzymes	All enzymes devoid of endo-1,4-β-xylanase (GH11) and α-l-arabinofuranosidase (GH43)	[all-(X_GH11 and_ A_GH43_)]
VII		All enzymes devoid of endo-1,4-β-xylanase (GH11) and α-l-arabinofuranosidase (GH51)	[all-(X_GH11 and_ A_GH51_)]
VIII		All enzymes devoid of endo-1,4-β-xylanase (GH10) and α-l-arabinofuranosidase (GH43)	[all-(X_GH10 and_ A_GH43_)]
IX		All enzymes devoid of endo-1,4-β-xylanase (GH10) and α-l-arabinofuranosidase (GH51)	[all-(X_GH10 and_ A_GH51_)]

The xylose release was monitored to evaluate the effect of the enzymes with different families each one endo-1,4-β-xylanase and α-l-arabinofuranosidase. For this analysis, a progress curve was divided into two stages. The first stage covered the monitoring of the reaction from time 0 to 90 min of reaction, taking samples at 10, 20, 40, 60, and 90 min, and the second stage continued the reaction for up to 48 h, taking samples after 4, 18, 24, and 48 h. Samples were heated at 100 °C for 10 min to stop the enzymatic reaction. All trials were performed in duplicate, and the mean difference was statistically assessed using Tukey’s test. Release xylose was determined by sugar analysis derivatized as alditol acetates (2.3.3), at least in duplicate. The xylose yield was calculated using Eq. 1:

1 $$\mathrm{Xylose\, yield} \left(\%\right)=\frac{\mathrm{ Xylose\, after \,enzymatic \,hydrolysis }(\mathrm{g})}{\mathrm{ Xylose \,in \,GAX \, fraction }(\mathrm{g})}$$

#### Sugar analysis after enzymatic hydrolysis

The released monosaccharides in supernatant enzymatic hydrolysis were analyzed as their alditol acetates by gas chromatography (Coimbra et al. 1996; Selvendran et al. 1979) using a FISONS 8340 chromatograph with a split injector (split ratio 1:60) and a FID detector. A DB-225 column (Agilent J and W, USA; 30 m × 0.25 mm × 0.15 Lm) was used. The injector and detector temperatures were 220 and 230 ℃, respectively. The oven temperature program started at 200–220 ℃ at a rate of 40 ℃ per min and was held at 220 ℃ for 15 min, then increased up to 230 °C with a rate of 20 ℃ per min and was held at 230 ℃ for 1 min. The flow rate of the carrier gas (H_2_) was set at 1 mL/min at 200 ℃. Using this technique, the xylose concentration is reported quantitatively, while the detection of arabinose was performed only qualitatively, reporting the presence or not of this sugar.

## Results and discussion

### Sugar and glycosidic-linkage composition of GAX extracted from BSG

**Fig. 1:**
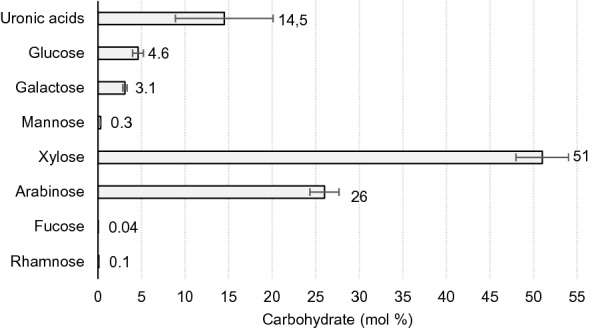
Carbohydrate composition of GAX fraction obtained by extraction of BSG with 4 M KOH

GAX fraction contains 889 g/kg of carbohydrates, mainly neutral sugars, and uronic acids. Figure 1 shows the sugar composition of GAX extracted in 4 M of KOH from BSG on a free base of proteins and phenolic compounds, because these compounds were not characterized in this work.

The carbohydrate was composed mainly of xylose (51% mol) and arabinose (26% mol). In this study, the quantification of uronic acids (14.5% mol) confirm the existence of glucuronoarabinoxylans in BSG, previously demonstrated qualitatively by Coelho et al. (2016) using electrospray ionization mass spectrometry (ESI-MS). The uronic acids amount found in this GAX were similar to those of steamed birchwood (11.5 mol %) (Kormelink and Voragen 1993). The GAX fraction of BSG also contains amounts of galactose (3.1% mol) and glucose (4.6% mol), this last probably due to the presence of residual starch (Reis et al. 2015) after the beer production. The remaining 11.1% (unknown) of the GAX surely corresponds to proteins, and phenolic acids. Proteins was already described to account 6–17% in AX extracted with 0.5 to 4 M KOH in BSG (Reis et al. 2015; Sajib et al. 2018). The presence of phenolic acids such *p*-coumaric and/or ferulic acid groups that could have remained esterified with arabinose in the branches of the main chain could also account with a small percentage to the extract; the values can range from 0.5 to 1.2 g/kg for ferulic acid and 0.1 g/kg for *p*-coumaric acid (Mandalari et al. 2005; Sajib et al. 2018).

**Fig. 2:**
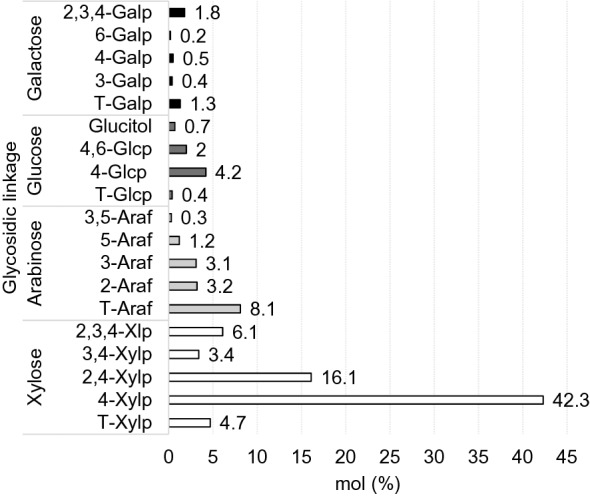
Glycoside linkage composition (mol %) of the GAX fraction obtained from BSG with 4 M KOH

According to part of the plant and the extraction method used, the AX fractions obtained can have different compositions, and consequently, their properties may differ (Biely et al. 2016; Bastos et al. 2018). Depending on the structural features of the xylan, different combinations of enzymes are required for maximum degradation (Kormelink and Voragen 1993). The structural features of GAX extracted from BSG were examined using methylation analysis (Fig. 2). Coelho et al. (2016) established the way to estimate the degree of polymerization (DP) of the AX is based on the principle that Xyl*p* does not occur as AX branching residues. On this assumption, the DP is obtained by the calculation of the relative amount of total xylose divided by the amount of terminally linked xylose, for the alkaline fraction used in this work the GAX from BSG was mainly composed by a backbone of 15 residues. From the results of linkage analysis, it can be deduced that the general structure of the xylans from GAX alkaline extracted (4 M KOH) from BSG consists of a linear backbone of (1 → 4)-linked-d-xylopyranosyl units (Xyl*p*) (42.3%). The degree of xylan main chain substitution by l-Ara*f* and MeGlcA varies and depends heavily on the extraction conditions and part of the plant used (Biely et al. 2016). In this GAX fraction from BSG the branched regions of the xylan backbone had monosubstitutions at *O*-2 (16.1% mol), 2-Ara*f* linkage was 3.2% indicates that α-1,2-linkage of 4-*O*-methyl-d-glucuronosyl units MeGlcA to the main chain Xyl*p* is approximately the remaining 12.9%, highlighting the need to use an α-glucuronidase enzyme to achieve complete hydrolysis. Arabinose occupied the substitution in 3-Xyl*p* position (3.1%). GAX also have disubstitutions at the 2 and 3 positions (6.1% mol). A lower degree of branching for arabinose was found, Ara:Xyl = 0.51, but higher for ratio of MeGlcA:Xyl = 3.57, showing an increase of this acid substitutions in the structure of the alkaline fraction extracted. Value similar was reported by Mandalari et al. (2005) for the fraction of BSG treated with cold water, 4 °C, 2 h (3.72) but lower than reported for the fraction treated with KOH 4.0 M, 2 h (13.95), although the fractionation protocol made by Vieira et al. (2014) was not as extensive as that reported by Mandalari et al. (2005).

### Comparison of enzymatic synergistic interactions

**Fig. 3:**
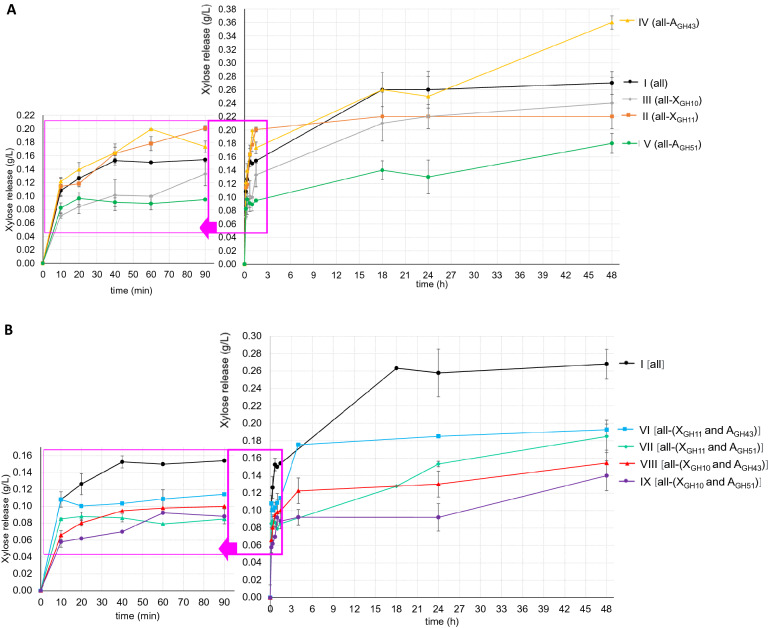
Xylose release from enzymatic hydrolysis. **A** Assays with all enzymes (I) versus assays without one enzyme (II to V). **B** Assays with all enzymes versus assays without two enzymes (VI to IX). Values represent the mean of two independent experiments, and error bars correspond to the standard deviations (SDs)

Nine scenarios were used to evaluate xylose release by cooperation of six purified enzymes (Table 1). In the first assay, a mix of all enzymes (I) was assessed. In assays II–V, one family enzyme (endo-1,4-β-xylanase or α-l-arabinofuranosidase) was devoid, and for assays VI–IX, two enzymes of one family of endo-1,4-β-xylanase and one family of α-l-arabinofuranosidase were eliminated simultaneously according to the definition in Table 2. Figure 3A shows the release xylose where Test I (all enzymes) is compared with assays where one endo-1,4-β-xylanase (test II and III) or one α-l-arabinofuranosidase (test IV to V) family enzyme was omitted throughout 48 h of reaction. In general, the highest xylose release 0.36 g/L achieved by the test IV (all-A_GH43_) after 48 h of reaction, was equivalent to a hydrolysis yield of 63.6%, whereas in test I (all), the yield was 52.9%. In assays II (all-X_GH11_), III (all-X_GH10_), and V (all-A_GH51_), the yield was below 50%.

These results highlight the stronger synergistic effect of GH51 over GH43 arabinofuranosidase family as debranching enzyme to obtain xylose from GAX of BSG and showed the synergism with α-glucuronidase GH67 added in the multi-enzymatic system evaluated. α-l-Arabinofuranosidase from family GH51 only attacks the (1→2) or (1→3) bonds on singly substituted xylopyranosyls and hence directly provides unsubstituted xylopyranosyls while α-l-arabinofuranosidase from family GH43 only attacks the (1→3) linked arabinose on doubly substituted xylopyranosyls. After removing the (1→3) linked arabinose, the β-xylosidase still cannot work on the xylan backbone, because the α-(1→2) linked arabinose blocks the binding site (Rasmussen et al. 2012). Moreover α-d-glucuronidase attacks the α-1,2 glycosidic bond between d-glucuronic acid or its ether 4-O-methyl-d-glucuronic acid from the terminal non-reducing d-xylose residues of xylo-oligosaccharides (aldo-uronic acids) and xylan. In turn this enhances the probability of more unsubstituted xylopyranosyls at (or near) the non-reducing ends for β-xylosidase to attack. Rasmussen et al. (2012) also compared the xylose release but from soluble wheat arabinoxylan for 4 h, at this time they found similar behavior from the combined treatments of xylanase + β-xylosidase + α-l-arabinofuranosidase GH51 (AF_An_) and the total combination of xylanase-β-xylosidase-α-l-arabinofuranosidase GH51 (AF_An_) + α-l-arabinofuranosidase GH43 (AF_Ba_). In this work a similar pattern was obtained up to 24 h between test I (all) and IV (all-A_GH43_), but after 24 h increased xylose release in the assay IV due to the presence of one α-d-glucuronidase of the family GH67. Long et al. (2022) also noted, that supplementation of the GH67 α-glucuronidase significantly enhanced (by 20–40%) the liberation of xylose from arabinoxylan of corncob during the enzyme hydrolysis (24 h), suggesting that MeGlcA substitution as an important limit factor of enzymatic saccharification remained in the substrate after the alkaline hydrogen peroxide pretreatment.

Figure 3A also shows the details of the first 90 min of the reaction in the left part of the figure. In these first 90 min of reaction, assays I (all), III (all-X_GH10_), IV (all-A_GH43_), and V (all-A_GH51_) showed a release of about 50% of the maximum xylose. Only assay II (all-X_GH11_) showed a release of 50% of the maximum xylose in less time (~ 10 min of reaction). The elimination of enzymatic activity related to endo-1,4-β-xylanases and α-l-arabinofuranosidases influenced the xylose yield over time. The results obtained showed that eliminating one of the endo-1,4-β-xylanases (GH10 or GH11 family) had different xylose release in the initial reaction rate (90 min). However, after 18 h and up to 48 h of reaction, assays II and III revealed no significant differences, showing the cooperation between these 2 kinds of xylanases. For the arabinose release presence of arabinose was evidenced at the first 90 min of the reaction for the assays I, II, III and IV, while for the assay V was not evident the arabinose presence corroborating the action of A_GH51_ not only to xylose release, but also in the arabinose release. Rasmussen et al. (2012) shows that the addition of xylanase and β-xylosidase each contributed to increase the arabinose release to different extents. However, neither xylanase nor β-xylosidase alone catalyzed the release of arabinose without the presence of arabinofuranosidase.

Figure 3B shows the xylose release in assays VI to IX, which omitted one endo-1,4-β-xylanase and one α-l-arabinofuranosidase simultaneously, compared with assay I (all enzymes present). The highest xylose release was 0.27 g/L achieved for assay I was equivalent to a hydrolysis yield of 52.9% compared with assays VI [all-(X_GH11 and_ A_GH43_)] and VII [(all-(X_GH11 and_ A_GH51_)], the yield for which was ~ 40%, whereas for assays VIII [all-(X_GH10 and_ A_GH43_)] and IX [all-(X_GH10 and_ A_GH51_)], the yield was ~ 30%. The results confirmed that the GH10 endo-1,4-β-xylanase family releases more xylose than the GH11 family; nevertheless, when they are at the same time this effect is not evident. The effect of eliminating GH43 or GH51 α-l-arabinofuranosidase families was less evident because the elimination of an endo-1,4-β-xylanase family was more important in the reaction. Rasmussen et al. (2012) inclusive indicated that the breaking down of the xylan backbone to smaller oligosaccharides, and thus exposing more (unsubstituted) non-reducing ends for β-xylosidase, is more important than removing the substituted arabinose to release more xylose from arabinoxylan.

The maximum yield (63.6%) reached in this work with the assay IV (endo-xylanase GH10 + endo-xylanase GH11 + α-L-arabinofuranosidase GH51 + α-glucuronidase GH67) is comparable to other arabinoxylan hydrolyzed enzymatically (Rasmussen et al. 2012; Long et al. 2022), but also show the necessity of supply the enzyme supplement with other accessory enzymes such as feruloyl esterase and α-l-galactosidase (Forssell et al. 2008; Mccleary et al. 2015; Biely et al. 2016; Long et al. 2022) and with other families of the α-glucuronidase such a GH115 to try increase the xylose release. Finally, due to the relevance of α-glucuronidase GH67 activity in the xylose release, it is important to probe other conditions of temperature and pH closest to the pH and temperature optima of this key enzyme.

## Conclusion

This research studied the action of four debranching and depolymerizing hydrolytic purified enzymes on GAX from BSG, quantifying the xylose released in the process. An enzyme mixture of two endo-xylanases (from the GH10 and GH11 families), α-l-arabinofuranosidase GH51, β-xylosidase GH43, and α-d-glucuronidase GH67, showed the best cooperation, with 63.6% maximum xylose yield in 48 h (40 °C, pH 5.5). Although α-d-glucuronidase shows a key cooperation in the multi-enzymatic system evaluated, it is also recommended to use a feruloyl esterase and galactosidase to increase the yield of xylose production in futures studies.

## Data Availability

Not applicable.

## Abbreviations

Ara*f*: L-Arabinofuranosyl residue
AX: Arabinoxylan
BSG: Brewers’ spent grain
DP: Degree of polymerization
EH: Enzymatic hydrolysis
ESI-MSn: Electrospray ionization mass spectrometric detection
GAX: Glucuronoarabinoxylan
GC-FID: Gas chromatography flame ionization detection
GC–qMS: Gas chromatography quadrupole mass spectrometry
GlcA: D-Glucuronic acid
MeGlcA: 4-O-Methyl-d-glucuronic acid
WAX: Wheat flour arabinoxylan
XOS: Xylooligosaccharides
Xyl: Xylose
Xyl*p*: D-Xylopyranosyl residue
